# Bridging data silos to holistically model plant macrophenology

**DOI:** 10.1111/nph.70249

**Published:** 2025-06-06

**Authors:** Lizbeth G. Amador, Tadeo H. Ramirez‐Parada, Isaac W. Park, Susan J. Mazer, Aaron M. Ellison, Margaret O'Brien, Eric R. Sokol, Colin A. Smith, Charles C. Davis, Sydne Record

**Affiliations:** ^1^ Department of Wildlife, Fisheries, and Conservation Biology University of Maine Orono ME 04469 USA; ^2^ Maine Agricultural and Forest Experiment Station Orono ME 04469 USA; ^3^ Department of Ecology, Evolution and Marine Biology University of California Santa Barbara CA 93106 USA; ^4^ Department of Biology Georgia Southern University Statesboro GA USA; ^5^ Department of Organismic and Evolutionary Biology Harvard University Herbaria 22 Divinity Avenue Cambridge MA 02138 USA; ^6^ Sound Solutions for Sustainable Science Boston MA 02135 USA; ^7^ Marine Science Institute, University of California Santa Barbara CA 93111 USA; ^8^ National Ecological Observatory Network, Battelle Boulder CO 80301 USA; ^9^ Center for Limnology, University of Wisconsin Madison WI 53706 USA

**Keywords:** data harmonization, data management, ontologies, scales, SDMs

## Abstract

Phenological response to global climate change can impact ecosystem functions. There are various data sources from which spatiotemporal and taxonomic phenological data may be obtained: mobilized herbaria, community science initiatives, observatory networks, and remote sensing. However, analyses conducted to date have generally relied on single sources of these data. Siloed treatment of data in analyses may be due to the lack of harmonization across different data sources that offer partially nonoverlapping information and are often complementary. Such treatment precludes a deeper understanding of phenological responses at varying macroecological scales. Here, we describe a detailed vision for the harmonization of phenological data, including the direct integration of disparate sources of phenological data using a common schema. Specifically, we highlight existing methods for data harmonization that can be applied to phenological data: data design patterns, metadata standards, and ontologies. We describe how harmonized data from multiple sources can be integrated into analyses using existing methods and discuss the use of automated extraction techniques. Data harmonization is not a new concept in ecology, but the harmonization of phenological data is overdue. We aim to highlight the need for better data harmonization, providing a roadmap for how harmonized phenological data may fill gaps while simultaneously being integrated into analyses.

## Introduction

Many biological interactions depend on phenological patterns that reflect ecological and evolutionary responses to climatic conditions (e.g. Chmura *et al*., 2019). For example, plant phenology – the recurring seasonal timing of leaf out, flowering, fruiting, and leaf senescence – is a key set of genetically and environmentally controlled traits that are central to plant reproduction, plant–pollinator interactions, and availability of resources to herbivores. Plant phenology is also linked directly to ecosystem processes and services relevant to human society, such as carbon sequestration, seasonal allergies, and food security (e.g. Fatima *et al*., 2020; Gray & Ewers, 2021; Cope *et al*., 2022). Because plant phenology is very sensitive to ongoing rapid environmental change, there is an urgent need to better quantify and predict plant phenological dynamics (e.g. Gallinat *et al*., 2021; D. S. Park *et al*., 2021), including how they pertain to species range changes (Peng *et al*., 2024; Ramirez‐Parada *et al*., 2025a).

A major challenge to quantifying and predicting phenology lies in its scale dependence (D. S. Park *et al*., 2021). Like many other ecological phenomena, inferences made about phenology depend on how data are combined across space, time, or taxa (Levin, 1992; D. S. Park *et al*., 2021). Furthermore, environmental drivers of phenology (e.g. temperature, precipitation, and insolation) can vary and interact differently across space and time (e.g. Peters *et al*., 2007; Chamberlain & Wolkovich, 2023), and plastic organismal responses to these drivers can differ among individuals, populations, species, and communities (Inouye *et al*., 2019; Ramirez‐Parada *et al*., 2024). Studying the effects of phenology on ecological processes at global scales requires data that span scales of time, taxonomy, and levels of biological organization. Understanding and analyzing scale dependencies of phenological responses is a growing field known as macrophenology, which can inform processes at larger spatial extents (Doi *et al*., 2017; Gallinat *et al*., 2021).

**Fig. 1:**
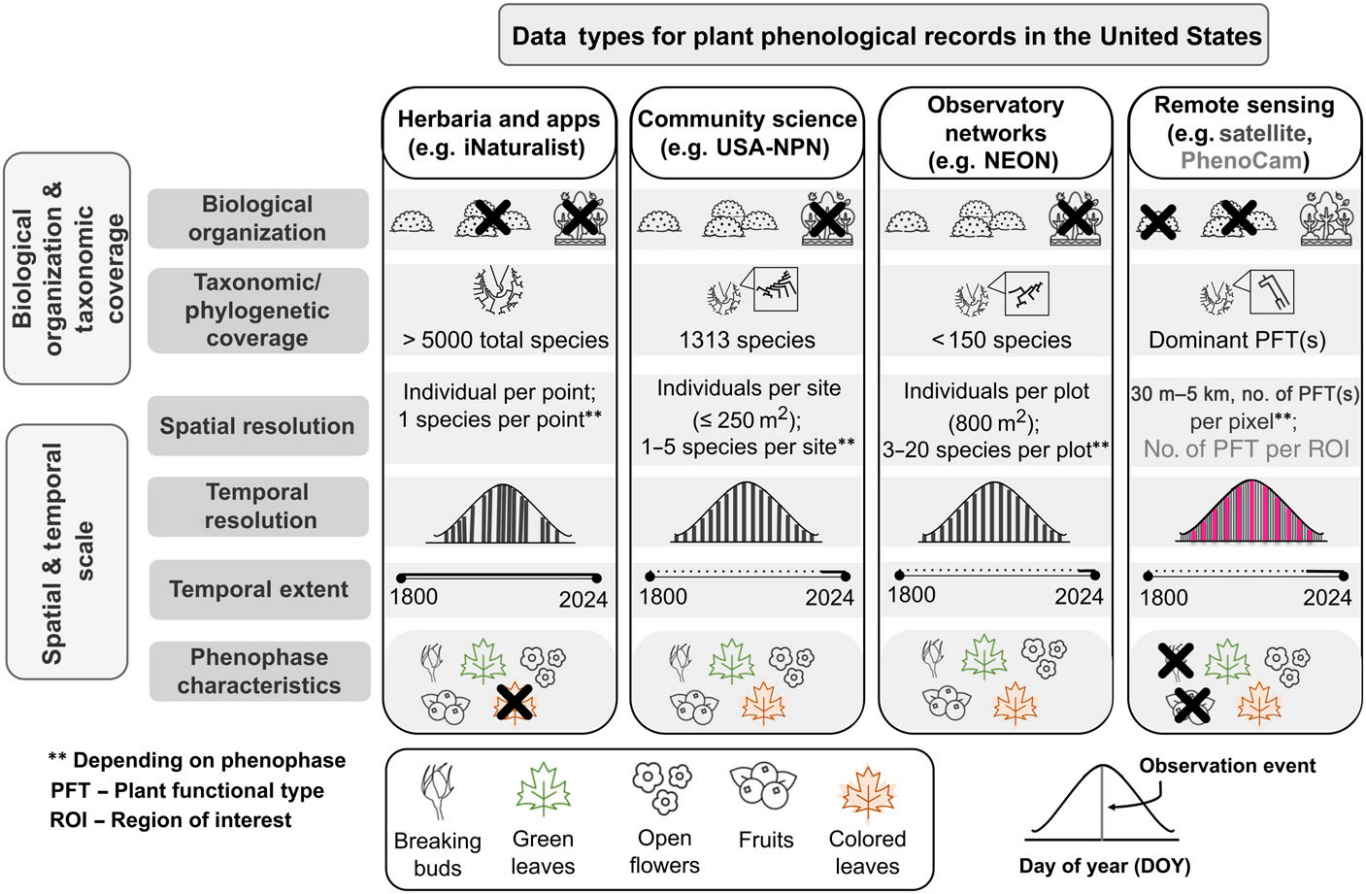
Data aspects across phenological data types with example datasets. Each data type offers a distinct level of biological, taxonomic, spatial, temporal, and phenophase information. Each level contains example‐specific information comparing the similarities and differences across data types. Differences between data types (across columns) highlight gaps where data harmonization would benefit and maximize coverage within each level. We use a bell curve to exemplify the sampling frequency within the duration of a phenophase. The bell curve in the remote sensing column exemplifies both satellite (bright red) and PhenoCam (grey). The color of their text also distinguishes the spatial resolution for satellites (dark grey) and PhenoCam (light grey). The young leaves symbol also includes fully opened green leaves for remote sensing and Phenocam, and the number of plant functional type (PFT) per region of interest (ROI) varies for Phenocams as the cameras capture different fields of view depending on landscape characteristics (e.g. topography; Liu *et al*., 2024). Note that there is an overlap between the National Ecological Observatory Network (NEON), community science, and remote sensing because NEON uses the data collection protocols of the National Phenology Network and the USA Phenocam Network, and the NEON Airborne Observation Platform collects remotely sensed hyperspectral and red‐green‐blue imagery.

Data sources across scales of space, time, taxonomy, and levels of biological organization do exist, although they have rarely been analyzed simultaneously. For plant phenological data, these include herbarium specimens, community science initiatives, observatory networks, and remote sensing platforms (e.g. Richardson *et al*., 2018; Gray & Ewers, 2021; Reyes‐González *et al*., 2021; Davis *et al*., 2022). Different data types capture disparate ecological levels and spatiotemporal scales as a result of their sampling design and effort. For instance, remote sensing may provide continuous landscape‐level monitoring over a long period of time, whereas observatory networks may provide periodic sampling with field surveys of individuals and populations that vary in their temporal extent (Fig. 1). These scale mismatches often hinder data harmonization – the direct integration of disparate data types under a common schema.

The lack of harmonization across datasets limits our ability to assess phenological responses to climate at various scales. All phenological data sources have blind spots on the ecological levels, generating observed variation across these levels. For example, remotely sensed data cannot resolve species; population‐level metrics (e.g. peak flowering dates) do not resolve individuals, and data from herbarium specimens and community science platforms (e.g. iNaturalist) are rarely repeated within individuals and populations. Integration of different phenological data sources can capture greater variation across scales. For instance, Iwanycki Ahlstrand *et al*. (2022) found that observatory network, herbarium, and remotely sensed data detected different temporal and spatial variation in phenology, such that combining these datasets captured greater variation. In some instances, data integration that captures greater variation across space leads to discoveries about regional differences in phenological responses. Everingham *et al*. (2023) used historic field data, herbarium records, and contemporary field data across New South Wales, Australia, and detected a delay in flowering phenology through time in the Southern Hemisphere compared with the advancement of flowering phenology in the Northern Hemisphere. Furthermore, single‐source analyses cannot directly assess how variability at one ecological level scales to determine patterns at the next level, making them less ecologically informative. For example, detecting a longer flowering season within a community is not enough to assert that the seasonal availability of flowering species is increasing: such lengthening could occur either because populations are flowering longer or due to a greater spread of flowering onsets among populations that may in fact decrease the average diversity of flowering species available throughout the season (Ramirez‐Parada *et al*., 2025b). Finally, although we emphasize here the value of cross‐scale analyses with data integration, it is also important to note that local extent studies provide invaluable data for local management and conservation.

In this viewpoint, we assert that data harmonization is critical for improving our understanding of the impacts of climate change on macrophenology (Melaas *et al*., 2016; Taylor *et al*., 2019; Gallinat *et al*., 2021; Iwanycki Ahlstrand *et al*., 2022; Everingham *et al*., 2023; Ramirez‐Parada *et al*., 2025a). We focus on plant phenological datasets collected predominantly in the United States, although similar data have been collected at sites world‐wide (Tsuchida *et al*., 2005, Nagai *et al*., 2010, Cook *et al*., 2012, Mariani *et al*., 2013, Davis *et al*., 2022, Domingo‐Marimon *et al*., 2022, Iwanycki Ahlstrand *et al*., 2022, D. S. Park *et al*., 2023; Supporting Information Table S1). We explore the characteristics of these data and reveal the unintentional data silos that limit our ability to answer a range of important ecological and evolutionary questions about phenology. Additionally, we identify how bringing together multiple data sources will enable us to answer new questions. To move toward a common goal of phenological data harmonization, we provide a road map describing methods for harmonization, how harmonization can help to fill gaps in phenological data across space and time, and methods for integrating harmonized data into analyses. We end with a call for harmonization of phenological data to rapidly advance phenological research.

## A multiplicity of data sources with different strengths and weaknesses

Phenological data have provided invaluable insights into the varying effects of changing climate on plants (Li *et al*., 2019; Zohner *et al*., 2023), and recent papers highlight potential new insights to be made from each independent data type (Davis *et al*., 2022; Dronova & Taddeo, 2022; Binley & Bennett, 2023; Zhu & Song, 2023). We cannot get a complete picture of phenology without integrating across data types due to gaps within any single data type. In this section, we draw attention to the strengths, weaknesses, and gaps of each data type with respect to space, time, taxonomy, life history, and levels of biological organization.

### Herbarium specimens

Each herbarium specimen provides phenological information observed at a specific historical point in time and at a specific location, and therefore reflects an individual plant's phenological response to recent or to long‐term climatic conditions. Collectively, herbarium specimens have been mobilized to study many species' and communities' phenological sensitivity to local climatic conditions and to climatic change (e.g. Davis, 2023) at broad spatial scales (e.g. Willis *et al*., 2017; Park *et al*., 2018; I. W. Park *et al*., 2021; Zhu & Song, 2023; Ramirez‐Parada *et al*., 2025b). Many of the large herbarium collections in the United States have increased the accessibility of phenological data contained in these specimens through massive efforts to digitize millions of physical specimens and the information contained in their labels with the centralization of data into repositories (e.g. the Global Biodiversity Information Facility (GBIF), n.d.; Southwestern Environmental Information Network (SEINet); GBIF; SEINet Portal Network, 2023) (Hedrick *et al*., 2020; Phang *et al*., 2022). However, specimens in countries with less digital infrastructure in place are less accessible or less frequently digitized, leading to biogeographical biases in spatial coverage (Daru *et al*., 2018; Davis *et al*., 2022). Despite the large taxonomic coverage at the species level, herbarium specimens provide relatively coarse phenophase information, represent single ‘snapshots’ of phenology in space and time, and may exhibit sampling biases that make it unclear whether a specimen represents an early, median, or late observation relative to its source population (Ramirez‐Parada *et al*., 2022; Park *et al*., 2024; Schmidt *et al*., 2025; Fig. 1). Moreover, as specimens represent single observations of individuals distributed widely in space and time, variation in phenology among specimens represents both within‐ and among‐population differences. Thus, identifying the level of ecological organization associated with relationships between phenology and environmental variables – and the mechanisms underlying such relationships – requires careful statistical design and interpretation of results (Davis *et al*., 2015; Pearse *et al*., 2017; Ramirez‐Parada *et al*., 2024). Another limitation of herbarium‐derived data for use in phenological studies is the difficulty in identifying dates of occurrence for phenological phases other than flowering and fruiting for most species. Additionally, their patchy temporal and spatial coverage can generate sampling biases that may limit their use at global or local scales (Daru *et al*., 2018; Schmidt *et al*., 2025).

### Community science initiatives

Community science initiatives harness the power of volunteers to record phenological data across broad spatial extents while providing high‐resolution phenophase information from a variety of taxa (Reyes‐González *et al*., 2021; Domingo‐Marimon *et al*., 2022). Such initiatives vary in the degree of standardization used in data collection. For instance, image contributors for community‐sourced app‐based records (e.g. iNaturalist) do not follow specific protocols for capturing phenophases. By contrast, the USA National Phenology Network (USA‐NPN) is an example of a community science initiative with volunteer engagement across the country through their Nature's Notebook platform (Crimmins *et al*., 2017; https://www.usanpn.org/). It has a standardized protocol to facilitate repeated observations of specific individuals (or patches) at a chosen site (Crimmins *et al*., 2017; Fig. 1). These data provide estimates of date of onset, termination, and duration of multiple phenophases at high temporal resolution and national coverage (Fig. 1). However, while these data encompass observations for thousands of species, most correspond to a narrow set of indicator species for which specific observational protocols have been developed. Furthermore, investment by volunteers leads to large variations in taxonomic coverage and duration of observations (e.g. a single vs multiple years). These data often contain observation bias and inconsistencies in protocol implementation that can limit their application (Reyes‐González *et al*., 2021; Domingo‐Marimon *et al*., 2022). In some cases, participants might record the phenological status of only one individual at one site many times per year (sometimes less), but they might not sample multiple individuals at a given site, thus greatly limiting population‐level inferences.

### Observatory networks

Observatory networks provide systematic, long‐term field data that follow individuals throughout their phenological cycle (Gallinat *et al*., 2021), thus providing opportunities to quantify inter‐ and intraspecific variation in phenology across ecoregions. The US National Science Foundation's National Ecological Observatory Network (NEON) is one such long‐term, ecological monitoring network designed to collect data through 2049 (Elmendorf *et al*., 2016; https://www.neonscience.org/). NEON works closely with the USA‐NPN and USA Phenocam networks to collect data and has adopted their standards and protocols (Richardson *et al*., 2007; https://phenocam.nau.edu/webcam/). This offers an exciting example of how different phenological monitoring systems can coordinate efforts for standardized observations and facilitate data harmonization (Richardson *et al*., 2007). Similar to NPN, NEON provides high‐resolution phenological information through repeated measures in their field sampling design (Fig. 1). NEON also records co‐located information on a suite of other biological and physical variables relevant to phenology (e.g. beetle pollinator abundance, climatic variables, and carbon dioxide flux; Nagy *et al*., 2021). Despite the continental scale and projected 30‐yr lifespan scale of NEON, their NPN‐style field observations are limited in (1) taxonomic coverage due to resource constraints; (2) spatial coverage as data are collected only at the several dozen established NEON sites; and (3) current temporal range due to the relatively recent establishment of the network in 2019 (Fig. 1). The Long‐term Ecological Research Network has some sites that collect phenological information, but these data are not collected with a standardized protocol, and synthesizing them is challenging (Mulder *et al*., 2021; Schulze, 2023; but see Keenan *et al*., 2014). Moving forward, NEON promises to be an irreplaceable long‐term reference for fine‐resolution phenological data compatible with all data amassed by the USA‐NPN through their active partnership and delivering NEON data through the USA‐NPN portal.

### Remote sensing

Remotely sensed data are capable of capturing continental and interannual changes in phenology, yet the spatial resolution of the data is often too coarse to discern phenological changes at the species level (Gallinat *et al*., 2021; Reyes‐González *et al*., 2021). Satellites, near‐Earth imagery, and PhenoCams have allowed for spatially continuous observations with increasing temporal resolution (Zarnetske *et al*., 2019; Lechner *et al*., 2020; Dronova & Taddeo, 2022; Latifi *et al*., 2023). Remote sensing techniques related to phenology have been applied successfully to detect the start and end of the growing season (i.e. leaf out and leaf off) for either dominant tree taxa or functional types, and primarily in temperate deciduous and tropical dry forests (Dronova & Taddeo, 2022). Recent applications of deep learning algorithms to high‐resolution hyperspectral and red‐green‐blue images (1 and 0.25 m, respectively) from near‐Earth (i.e. airborne) remote sensing of NEON sites enable the segmentation and identification of individual tree crowns (Weinstein *et al*., 2024), paving the way for the detection of individual tree‐crown phenology from frequent near‐Earth image acquisition (e.g. by drones). Furthermore, the National Aeronautics and Space Administration's Surface Biology and Geology High Frequency Time Series (SHIFT) near‐Earth remote sensing campaign in 2023 enabled the detection of superblooms in the grasslands of coastal California from weekly flyovers (Angel *et al*., 2025). Although hyperspectral sensors have broadened the possibilities of remotely sensed phenological monitoring, such efforts remain limited to specific sites (e.g. NEON sites) or campaigns with high spatial and temporal resolution. Another limitation is that the earliest remote sensing data are limited to the 1970's (i.e. Landsat 1 products) and do not provide substantial preglobal warming information comparable to point‐based herbarium data.

## Moving forward: bridging data silos in macrophenology

### Data harmonization

**Fig. 2:**
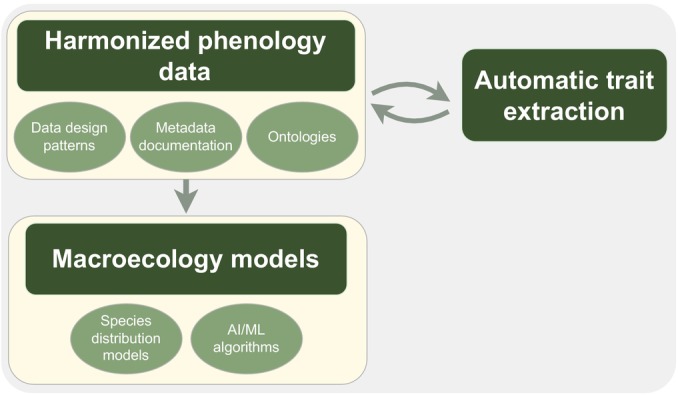
Cyclical connections between phenological data harmonization and artificial intelligence (AI)/machine learning (ML) trait extraction can produce data to feed into models, further supporting improved predictions of plant phenology.

Data harmonization is not a new concept in ecology. For decades, there has been tremendous interest across the scientific community in pooling and harmonizing plant trait data (Keune *et al*., 1991; Tarboton *et al*., 2008; Reichman *et al*., 2011; Wieczorek *et al*., 2012; Boyle *et al*., 2013; Pollet *et al*., 2015; Stucky *et al*., 2018; Record *et al*., 2021; Flantua *et al*., 2023). For instance, the TRY database has excelled in aggregating trait data and supported extensive advances in trait‐based plant ecology, but lacks a common format that limits compatibility between data sets (Kattge *et al*., 2011, 2020). More recently, ecologists have recognized the importance of considering intraspecific trait variation, emphasizing the coordination of open science efforts around individual‐level trait information (Violle *et al*., 2012; Cope *et al*., 2022). Except by remote sensing networks, phenological data are collected from individual organisms and allow for exploration of intraspecific trait variation. This makes phenological data an excellent test bed for developing and testing approaches for data harmonization of individual‐level traits. Various approaches exist for harmonizing ecological data that could be applied to phenological data. Many of these approaches incorporate common terminology and structures (i.e. design patterns) representing relational tables tracking organismal information (e.g. taxonomy and measurement; i.e. trait, number of individuals) and other important metadata (e.g. geographic locations and differences in sampling methodologies; O'Brien *et al*., 2021; Keller *et al*., 2023; Fig. 2).

Using common terminology and notation is a key aspect of harmonizing phenological data to make it easier for researchers (and algorithms) to discover and use data. Ontologies provide a structured, formal language for the standardization of terminology and concepts related to data management (see Stucky *et al*., 2018; Schneider *et al*., 2019; Lenters *et al*., 2021; O'Brien *et al*., 2021; Dumschott *et al*., 2023; Keller *et al*., 2023). To our knowledge, the most well‐developed ontology of phenology terms is the Plant Phenology Ontology (PPO; Stucky *et al*., 2018), which assembled a robust aggregated vocabulary from global phenological records. Contributions toward such efforts are crucial for dispelling uncertainties in naming conventions for phenophases. For example, intensity‐based vocabulary may require a minimum percentage of reproductive organs to be displayed as ‘open flowers’ for an individual's phenophase to be identified as ‘flowering’, whereas qualitative assessments of flowering status may simply require the presence of a single open flower. PPO uses a framework that allows integration with vocabularies that capture other ecological traits and important information (e.g. the location of observation). The Ecological Trait Data Standard (ETS; Schneider *et al*., 2019) is a common vocabulary to facilitate trait data harmonization and is implemented into some trait data integration networks (Open Traits Network; Gallagher *et al*., 2020). Notably, ETS incorporates terms used by the Darwin Core Standard (DwC), a glossary of terms to facilitate sharing information about biodiversity maintained by the Biodiversity Information Standards Taxonomic Databases Working Group. Common vocabularies propagate a shared understanding of phenological phenomena that lay the groundwork for the harmonization of data into a common structure. Robust multilingual vocabularies already exist; ENVO (Buttigieg *et al*., 2016) and EnvThes (FAIRsharing.org, 2025), both describe ecosystem‐level concepts that dovetail with phenology and could be integrated at some level. PPO, ENVO, and EnvThes are all capable of handling languages coded by ISO 639‐3 (which includes Indigenous languages), although for the most part, translations are limited to major European languages and English. Work to reconcile subtle differences in meaning and cultural context is complex (Van Derbilt *et al*., 2010), and use of Indigenous languages will require additional extensive work in areas of governance and data sovereignty (Jennings *et al*., 2025).

**Fig. 3:**
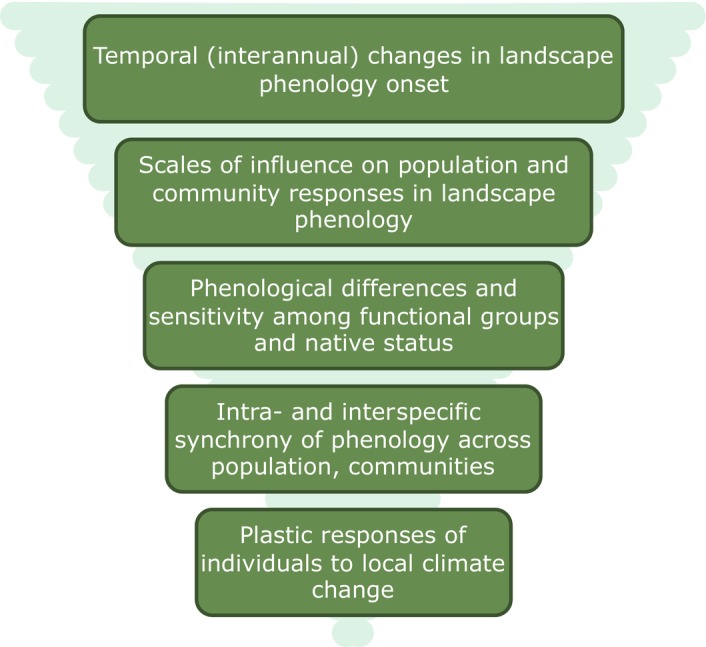
Hierarchy of general objectives in plant macrophenology, from broad to specific. The phenophase event of interest may determine the data types appropriate for achieving the objective.

Common terminology may also make it easier to consistently document differences in protocols for observing the phenophase across data types, which is important for contextualizing inferences from the data. For instance, when combining community science and observatory network data in analyses, it is incumbent to know the difference between observation methods because each has different levels of observation uncertainty (Binley & Bennett, 2023). Accessible information about sampling design would help compare levels of uncertainty between data types and assess their degree of interoperability for specific research objectives (Fig. 3). Sharing reproducible methods in open platforms, such as protocol.io (https://protocols.io; Accessed 3 June 2024) will contribute toward the development of standard disciplinary formats that are useful in metadata curation. Shared protocols between NEON and NPN are a notable example, but even their metadata are found only on site‐specific publications or their websites. Furthermore, any protocols used in the collection or curation of herbarium specimens (including their digital records) that are relevant to plant phenology should be included in the metadata or the specimen label. This fits perfectly with the concept of the Global Metaherbarium and the Extended Specimen concept (Davis, 2023). Protocol standards with accessible metadata information and sampling disclosures will be key in supporting data integration and harmonization among data sources while highlighting commonalities and differences in observations (Schneider *et al*., 2019; Dumschott *et al*., 2023; Keller *et al*., 2023).

**Fig. 4:**
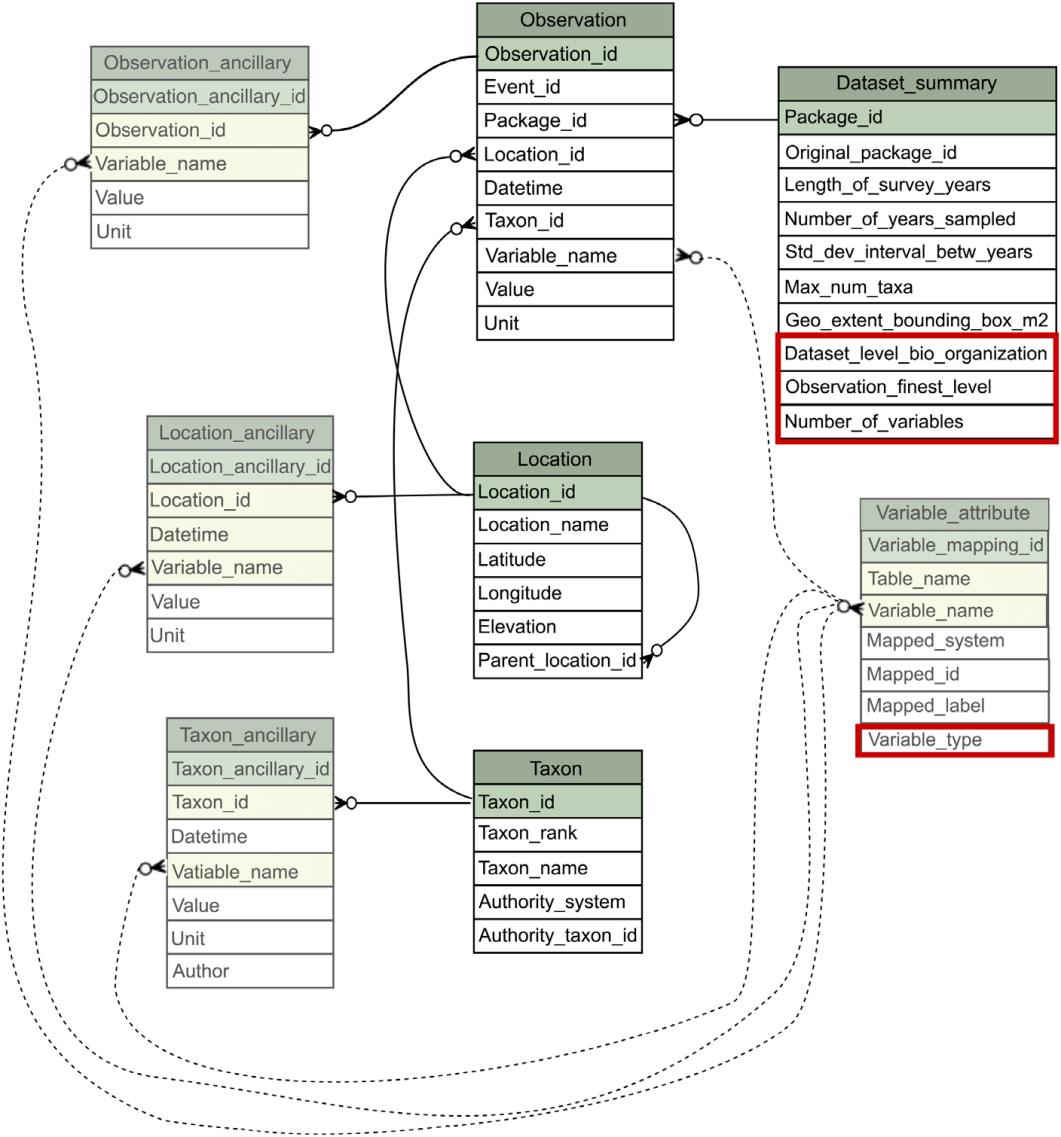
Schema of the updated ecocomDP data design pattern with the extension to accommodate traits (O'Brien *et al*., 2021). Added fields in the dataset summary table (boxed in red) allow users to include the level of biological organization, level of observation, and the number of variables associated with the trait. The variable attribute (previously variable_mappling) now includes variable type to indicate the type of trait measured (e.g. start and end dates).

Along with common terminology and well‐documented protocols for phenological data collection, a robust data design pattern (i.e. relational structure) will empower the increased integration of harmonized data into derived data products that may make it easier to account for differences in sampling effort or biases in downstream analyses. Although there are various data design patterns for plant traits (e.g. structural traits of palms incorporating the ETS; Lenters *et al*., 2021), there is not a well‐adopted data design pattern for plant phenological traits. We propose a phenological trait extension of the Ecological Community Data Design Pattern (ecocomDP), which was developed for harmonizing community ecology biodiversity data (O'Brien *et al*., 2021). The original ecocomDP model is extended with two features: (1) reconfiguring the table for mapping variables to external dictionaries to allow any variable attribute (e.g. a trait) to be recorded and linked to an external dictionary of concepts, such as the ontologies mentioned above; and (2) adding additional descriptive fields to the dataset summary table (Fig. 4, red boxes). Because ecocomDP already accommodates community‐level analyses, this extension would enable researchers to ask questions across levels of biological organization (e.g. from individuals to populations to communities). Another advantage of incorporating trait data into ecocomDP is that existing NEON and Long Term Ecological Research (LTER) Network data from various taxa are already harmonized with ecocomDP, making it a good candidate for the future incorporation of phenological traits and additional individual‐level traits. ecocomDP also employs concepts used by ETS and DwC, making data harmonized into its structure easily convertible to the DwC‐Archive. Finally, ecocomDP strongly emphasizes metadata, which is essential to ensure that downstream users can determine the relevance of the data for their study objectives through filtering.

Although ecocomDP is designed to harmonize *in situ* phenological observations, this initial step toward standardization across observations has the potential to increase the compatibility of *in situ* and remotely sensed phenological data. When harmonizing *in situ* and satellite phenological data, one must consider the spatial resolutions at which they are recorded (Angel *et al*., 2025). Many *in situ* phenological data are point observations or recorded on a designated plot. Remotely sensed phenological satellite data are gridded or rasterized information and may be recorded at different spatial, temporal, and spectral resolutions. Robust data design patterns like ecocomDP ensure that spatial metadata (e.g. latitude/longitude and geographic coordinate systems) for *in situ* observations are included in an intermediate format that is readily interoperable and can be further derived into gridded information for alignment with rasterized satellite images (O'Brien *et al*., 2021). The ecocomDP model is based on when‐where‐taxon‐what_was_measured. The location table is based on a point, which works for small plot data. For satellite data, a pixel is best represented with its center point as the locus, and its size, extent, and dimensions recorded in the location_ancillary table. Taxa are often inexact, and this is particularly true for satellite data. Exact identification is not required by the model. Indiscernible taxa can be listed as ‘Taxon 1’, ‘Taxon 2’, etc., with finer identification and taxonomic reference added later. A key value table accommodates any measurement. For satellite data, the simplest measurement might be color intensity, or some other measurement obtained via an RS algorithm, linked to an identifier with more information.

For instance, formatting phenological data from different *in situ* sources (e.g. herbaria, phenocams, and field observations) into a common intermediate format complete with spatial metadata (e.g. spatial point or bounding box coordinates with geographic coordinate system information) facilitates the integration of *in situ* phenological observations and satellite‐derived phenological data that may be recorded at different spatial resolutions. Phenological estimates derived from USA‐NPN or herbarium point observations can be summarized within the grid cells of a raster that is of the same resolution and extent as the rasterized image of phenological satellite data. For example, USA‐NPN creates spring indices to map the onset of spring based on observations submitted by community observers (Crimmins *et al*., 2017). Furthermore, climate data can be used to create anomaly indices of events or near‐term forecasts, such as the date of the first appearance of leaves or flowers, similar to the start of season and end of season satellite‐derived products (Schwartz & Hanes, 2010; Crimmins *et al*., 2017; Wheeler *et al*., 2024). Derived products from data design patterns, such as ecocomDP, can streamline reformatting tasks between point and rasterized phenological data, facilitating compatibility between the two data sources.

### Methods for integrating harmonized phenological data into analyses

When modeling phenology, we need to expand our perspective on where we can apply observations beyond classic phenological models (i.e. location‐specific growing degree day models; Chamberlain & Wolkovich, 2023). With the integration of different data types into analyses, models must account for underlying biases from different data containing information on phenology and its drivers across spatial and temporal scales. Here, we discuss macroecological approaches to solving two challenges: (1) differences in how phenophases are recorded; and (2) spatial and temporal mismatches between phenological, geographic, and climatic data. These hurdles greatly impede efforts of macroscale phenology studies as they limit the geographic scope and questions that may be explored (Gallinat *et al*., 2021).

To address the first challenge of differences in how phenophases are recorded, there are many methods phenologists could adapt from species distribution modeling (SDM). Instead of modeling a response of species occurrence or abundance, we can model the probability of occurrence of a phenophase throughout the year. For instance, such a model could be used to create rasterized forecasts of species‐level phenological point observations from herbarium, community science, or observatory network data into a spatially gridded dataset that is compatible with remote sensing data (Peng *et al*., 2024). For example, Yoder *et al*. (2024) used herbarium and community science data to create gridded predictions of whether Joshua trees are expected to have masted or flowered for each year and location. These rasterized predictions of mast events can then be compared with remotely sensed gridded data on leaf phenology (i.e. peak greenness). Notably, most airborne or satellite remotely sensed phenological data cannot discern information below the community level (e.g. plant functional types), but combining such remotely sensed data with harmonized *in situ* phenological observations (e.g. point data for dominant genera or species from site visits or PhenoCams) can enable inference of higher taxonomic resolutions (see Browning *et al*., 2017; Chandra *et al*., 2022; Domingo‐Marimon *et al*., 2022; Shao *et al*., 2023; Angel *et al*., 2025).

Another approach to account for differences in how phenophases are recorded is occupancy modeling (OM), which is widely used in the field of macroecology to model species distributions and provides a rich methodology by addressing imperfect detection and incorporating geographic location error from specimens for analyses of harmonized phenological data with differences in sampling effort (Erickson & Smith, 2021). One difference in sampling effort presented by phenological data is that some data types only record presence‐only (PO) information on phenophases (e.g. herbarium records; iNaturalist), whereas others record both presences and absences (PA) of phenophases (e.g. NPN and NEON). With an OM framework, differences in sampling effort can be accounted for by treating each data type as a designated survey or method to account for differences in detection. Recent advances with integrated SDMs that model PO and PA responses provide a powerful approach for combining data types with differences in sampling effort (Miller *et al*., 2019; Isaac *et al*., 2020; Mäkinen *et al*., 2024). Integrated SDMs could be a powerful way to combine disparate phenological data types (Box 1) and overcome the challenge of accounting for differences in how phenophases are recorded.

Box 1 **Integrated Species Distribtuion Model (ISDM) roadmap for harmonized models**

To illustrate how ISDMs could be used to simultaneously model different types of phenological data, we present a roadmap to constructing a harmonized model using open flowers of red maples (*Acer rubrum*) using open‐source data types that differ in detection of flowering (herbarium (PO) and field observations from National Ecological Observatory Network (NEON) and USA National Phenology Network (USA‐NPN) (PA)). To better understand the heterogeneity across data types, it is important to first understand where observations occur in space in time to guide model parameterization (panel i, ii). Visualizations of observations for each data source depict disparities in sampling effort across time. The main assumption for an ISDM is that the data observed are modeling the same ecological state where the true distribution is unknown (i.e. latent state; panel iii). The common parameters shared between each model address the assumption of observations pertaining to the same ecological state (panel iv). The ISDM incorporates shared spatial biases and any known sampling biases into the species‐specific models to predict the probability of flowering occurring across space at a point in time (panel iv) to produce a probabilistic map of flowering across space at that time. Examples of available software for running ISDMs include the pointedSDM or intSDM R packages (Mostert & O'Hara, 2025; Mostert *et al*., 2025).

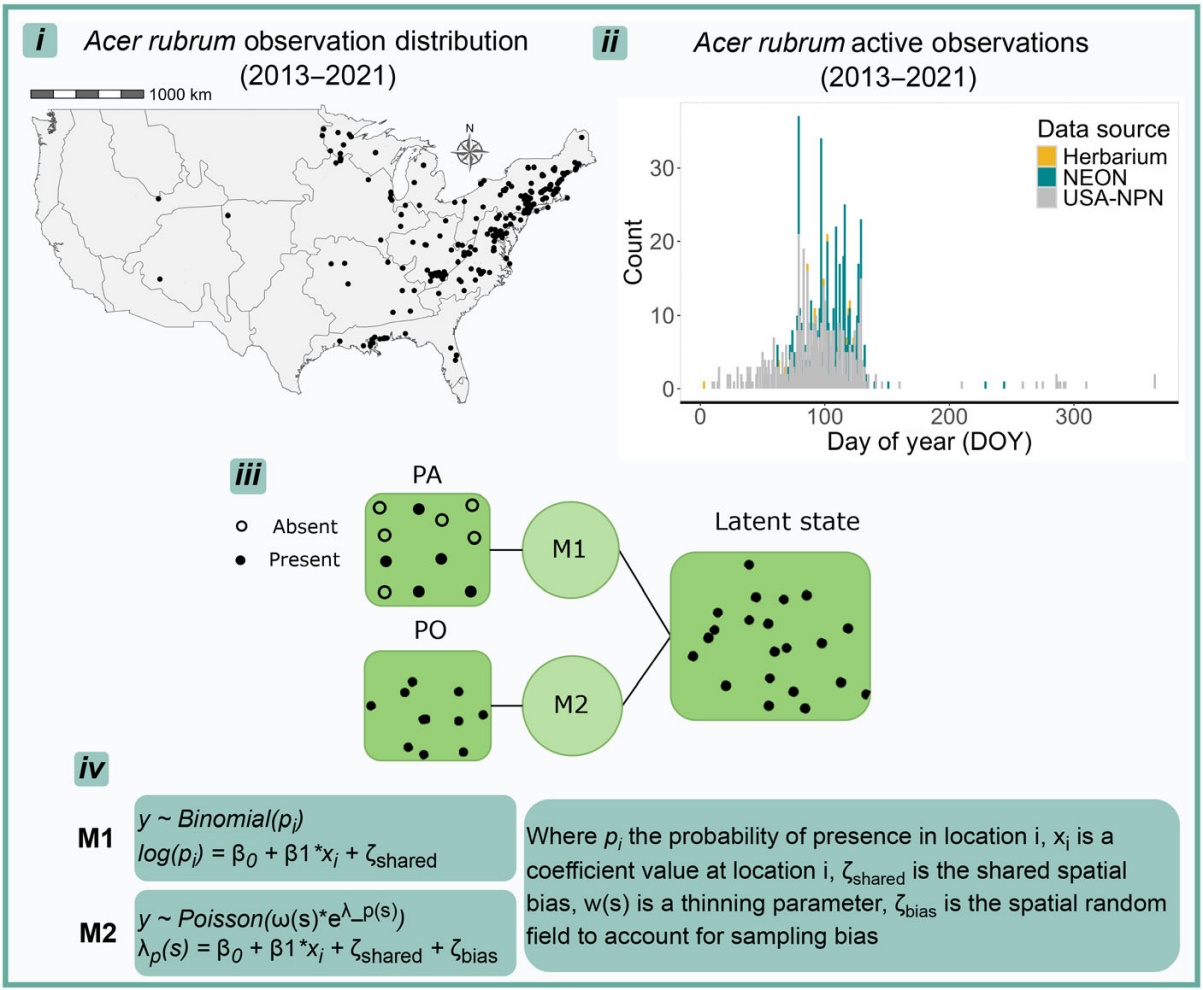

A second challenge is that phenological data and drivers of phenological responses are often measured at different temporal and spatial scales (and therefore, levels of biological organization; Fig. 1). Automated integration and synthesis tools have begun to be developed to facilitate cross‐scale phenological studies. For instance, the Pheno‐Synthesis Software Suite (PS3) summarizes ground‐based phenological observations into gridded climate and phenological indices (Morisette *et al*., 2021). One consideration in using such software is understanding what spatial and temporal resolutions and extents have the greatest influence on phenology: to explore the optimal spatial scale between phenological data and its drivers (e.g. climate and land use topography), different grains (e.g. varying radii around a central phenological observation point or pixel) and extents (e.g. continental, ecoregion, and site for NPN or NEON) that are then compared in analyses (Zarnetske *et al*., 2019; Read *et al*., 2020; Li, 2022).

A promising approach for exploring scales of space and time simultaneously is through interpretable machine learning (ML; see Box 2). Interpretable ML aims to understand what input data characteristics are most important in driving predictions of output data. Local interpretation with ML – wherein the prediction of a model for a single observation in space and time is considered, as opposed to trying to understand the overall predictive behavior of the model across the entire dataset – is especially relevant for exploring spatiotemporal drivers of geo‐referenced phenological data. This allows for the visualization and estimation of interactions between location features (i.e. spatial coordinates of phenological data points or grid cells) and other model features (e.g. temperature data represented by different spatial resolutions or temporal lags; Li, 2022). An example of a local interpretation method comes from an extension of the Shapley value in game theory (Shapley, 1953), which evaluates how contributions of different players collectively result in a contest's outcome. SHapley Additive exPlanations (SHAP), a recent ML offshoot of Shapley values, quantifies how much each feature collectively contributes to averaged model predictions (Štrumbelj & Kononenko, 2014). Historical phenological data could be used as features in such a model to predict contemporary or future phenological responses. The Shapley value and other local interpretability methods (e.g. Local Interpretable Model‐agnostic Explanation; Ribeiro *et al*., 2016) offer an exciting new opportunity to simultaneously explore spatiotemporal effects of drivers of harmonized phenological data. Overall, existing modeling approaches from macrosystems ecology and data informatics pose unique solutions to challenges by the integration of phenological data simultaneously into analyses.

Box 2 **Glossary**

**Data design pattern:** a blueprint that captures the essential data characteristics so that a centralized workflow can access, reformat, and structure data (O'Brien *et al*., 2021).

**Data harmonization:** direct integration of different plant phenological data categories (e.g. community science, herbarium, and remote sensing) under a common schema.

**Data integration (or data interoperability):** disparate data sources that may be used in tandem and are readily applicable in modeling or management frameworks (Wilkinson *et al*., 2016; Stucky *et al*., 2018; Brenskelle *et al*., 2019).

**Data management:** the organization and handling of data that supports its continuous discovery, evaluation, and reuse (Wilkinson *et al*., 2016).

**Machine learning (ML):** a subset of methodologies that use algorithms to automate learning predictions about data (e.g. Deep Learning, random forest; ‘Artificial Intelligence (AI) vs Machine Learning’, n.d.; Pearson *et al*., 2020).

**Occupancy model (OM):** a spatially explicit model that determines the occupation of an organism using presence and absence information.

**Ontology:** standardized vocabulary and a language framework using formal logic that relates terms to concepts and allows for the integration of different data (Madin *et al*., 2008; Stucky *et al*., 2018).

**Plant phenology:** the timing of recurring life stages (reproductive or growth) of a plant; with a focus on angiosperms.

**Phenophase:** the phenological stage of a plant or animal that details a particular life cycle stage (e.g. leaf emergence, migration, and breeding).

**Species distribution modeling (SDM):** a form of occupancy modeling that predicts species distributions over space based on the attributes of the locations where they are currently known to occur.

## Concluding remarks

Predicting plant phenological responses to global change is important given its close ties to ecosystem processes and human health. However, given the scale dependence of plant phenology, it is difficult to make informed predictions in the absence of data that spans space, time, taxa, and levels of biological organization. Fortunately, such data are at our fingertips through various efforts in recording plant phenology at different scales and with different methods of observation, but the distinct types of phenological data need to be harmonized to unlock their full potential. Efforts to bridge phenological data silos can benefit from successful examples from other subdisciplines in ecology. Approaches to harmonize data can be adopted from existing ecological data design patterns, metadata standards, and ontologies. Biogeographic and macroecological studies offer many solutions for integrating disparate data with unique sampling biases into models. They provide a rich methodology for tackling imperfect detection and incorporating geographic location error from specimens (Erickson & Smith, 2021). Data informatics approaches are another promising tool to automate data extraction and harmonization while improving predictions of plant phenology through pattern detection. Data interoperability is not a new concept in ecology, and phenological data harmonization is long overdue.

## Competing interests

None declared.

## Author contributions

LGA, AME, and CCD conceived the initial ideas which were further developed and refined with SJM, IWP, THR‐P and SR. LGA designed and developed outlines which were further refined by SR, CCD, AME, IWP, SJM, and THR‐P. LGA and MO'B drafted and compiled figures which were further refined by SR, CCD, AME, IWP, SJM, THR‐P, CAS, and ERS. LGA led the writing of the manuscript. SR, CCD, AME, IWP, SJM, MO'B, CAS and ERS contributed significantly to the subsequent revisions. SR served as PhD advisor for LGA All authors contributed critically to the drafts and gave final approval for publication.

## Disclaimer

The New Phytologist Foundation remains neutral with regard to jurisdictional claims in maps and in any institutional affiliations.

## Supporting information


**Table S1** Nonexhaustive list of global plant phenological networks with summaries of their datasets.
Please note: Wiley is not responsible for the content or functionality of any Supporting Information supplied by the authors. Any queries (other than missing material) should be directed to the *New Phytologist* Central Office.

## Data Availability

The data used to create graphs from Box 1 are openly available in Environmental Data Initiative (EDI) at doi: 10.6073/pasta/569c592db3a875f084e7761ff66844d9, package ID: edi.1995.1. Additionally, the data derived are available from USA National Phenology Network at doi: 10.5066/F78S4N1V, National Ecological Observatory Network (NEON) at https://www.neonscience.org/data, Dryad at https://datadryad.org/stash, and EDI at https://edirepository.org/. These data were derived from the following resources available in the public domain: Switzer *et al*. (2024), NEON (2024), I. Park *et al*. (2023), D. Park *et al*. (2023).
