# Supplementary material for: Bridging data silos to holistically model plant macrophenology
Source: New Phytol. 2025 Jun 6;251(2):625–36. doi: 10.1111/nph.70249 (PMC13278655; doi:10.1111/nph.70249)
Supplement: Supplementary file 1 — Table S1 Nonexhaustive list of global plant phenological networks with summaries of their datasets. Please note: Wiley is not responsible for the content or functionality of any Supporting Information supplied by the authors. Any queries (other than missing material) should be directed to the New Phytologist Central Office. [file NPH-251-625-s001.docx]

***New Phytologist* Supporting Information**

Article title: Bridging data silos to holistically model plant macrophenology

Authors: Lizbeth G. Amador^1,2^, Tadeo H. Ramirez-Parada^3^, Isaac W. Park^3,^ ^4^, Susan J. Mazer^3^, Aaron M. Ellison^5,6^, Margaret O'Brien^7^, Eric R. Sokol^8^, Colin A. Smith^9^, Charles C. Davis^5^, Sydne Record^1,2^

^1^Department of Wildlife, Fisheries, and Conservation Biology, University of Maine, Orono, ME 04469, USA

^2^Maine Agricultural and Forest Experiment Station, Orono, ME, 04469, USA

^3^Department of Ecology, Evolution and Marine Biology, University of California, Santa Barbara, CA, 93106 USA

^4^Department of Biology, Georgia Southern University, Statesboro, GA, USA

^5^Department of Organismic and Evolutionary Biology, Harvard University Herbaria, 22 Divinity Avenue, Cambridge, MA 02138, USA

^6^Sound Solutions for Sustainable Science, Boston, MA 02135, USA

^7^Marine Science Institute, University of California, Santa Barbara, CA 93111, USA ^8^National Ecological Observatory Network, Battelle, Boulder, CO 80301, USA ^9^Center for Limnology, University of Wisconsin, Madison, WI 53706, USA

Article acceptance date: 6 May, 2025

The following Supporting Information is available for this article:

**Table S1.** Non-exhaustive list of phenology networks across the globe. Here we attempted to summarise the scope of plant phenological data in various regional networks across space, time, data type, plant functional types, phenophase measured, and a hyperlink to the website. A handful of networks did not have a website accessible to the authors, so we hyperlinked a paper discussing the network instead. Note the different terms used for plant functional types and phenophases across networks. These differences in terminology were maintained to depict the differences in terms related to plant functional types and phenophases for various reasons (e.g., language, and discipline of the primary investigators).

| **Organization** | **Country of origin** | **Geographic coverage** | **Temporal Extent** | **Data type** | **Plant Functional Types** | **Phenophases** | **Website** |
| --- | --- | --- | --- | --- | --- | --- | --- |
| USA National Phenology Network | United States | United States, Canada, Puerto Rico | 2008-Present | Community Science | Varies | breaking leaf buds, leaves, increasing leaf size, colored leaves, falling leaves, flowers or flower buds, open flowers, fruits, ripe fruits, recent fruit or seed drop; pollen release | [USA-NPN](https://www.usanpn.org/home) |
| CrowdCurio | United States | Eastern United States | Varies | Herbaria | Varies | flower, leaf, buds, fruit | [CrowdCurio](https://nph.onlinelibrary.wiley.com/doi/10.1111/nph.14535) |
| Network of Ecological and Climatological Timings Across Regions | Canada | United States, Canada, United Kingdom | 1739-2010 | Mixed | Varies | first flowering, first leafing, first leaf bud, full leaf | [NECTAR](https://knb.ecoinformatics.org/view/nceas.988.17) |
| iNaturalist | United States | Global | 2008-Present | Crowd-sourced herbaria -- app-based | Varies | Varies | [iNat](https://www.inaturalist.org/) |
| Pan European Phenology Project 725 | Austria | Austria, Andorra, Norway, Belgium, Poland, Croatia, Romania, Czech Republic, Serbia, Finland, Slovak Republic, Germany, Slovenia, Hungary, Spain, Ireland, Sweden, Macedonia, Switzerland, Montenegro, France, Italy, Bosnia and Herzegovina, Bulgaria, Denmark, Greece, Latvia, Lithuania, Luxembourg, Netherlands, Portugal, Romania, Ukraine, United Kingdom | 2011-2025 | Community science | Varies | start of flowering, full flowering, fruit softening, fruit ripen, first true leaf, first ripe fruits, dry seed, start of harvest, end of flowering, autumnal leaf colouring, winter dormancy | [PEP 725](http://www.pep725.eu/) |

| National Ecology Observation Network | United States | United States, Puerto Rico | 2013-Present | Plot surveys | Varies | breaking leaf buds, leaves, increasing leaf size, colored leaves, falling leaves, flowers or flower buds, open flowers, fruits, ripe fruits, recent fruit or seed drop; pollen release | [NEON](https://www.neonscience.org/) |
| --- | --- | --- | --- | --- | --- | --- | --- |
| USDA Forestry Inventory and Analysis | United States | United States |  | Plot surveys | Varies | Varies | [USDA FIA](https://research.fs.usda.gov/programs/fia) |
| Pl@ntNet | France | Global | 1970-2023 | Crowd-sourced herbaria -- app-based | Varies | Varies | [Pl@ntNet](https://identify.plantnet.org/) |
| Himalayan Environmental Rhythms Observation and Evaluation System | Bhutan | Bhutan | 2015-present | Community Science | herbaceous, shrubs, deciduous trees, coniferous trees | budding, leafing, flower buds, open flowers, fruiting, ripe fruits, leaf fall, seeding | [HEROES](https://heroes.uwicer.gov.bt/about) |
| budburst | United States | United States, Puerto Rico | 2011-2023 | Crowd-sourced herbaria -- app-based | forbs, deciduous trees, deciduous shrubs, broadleaf evergreens  , conifers, grasses | leafing, flowering, fruiting, leaf color, senescence | [budburst](https://budburst.org/the-program) |

| Center for Tropical Forest Science - Forest Global Earth Observatory | United States | United States, Puerto Rico; Canada, Costa Rica, Panamá; Ecuador, Brazil, United Kingdom, Czech Republic, Germany, Nigeria, Cameroon, Gabon, DR of Congo, Kenya, India, Sri Lanka, China, Thailand, Vietnam, Taiwan, Philippines, Malaysia, Singapore, Palau, Papua New Guinea | 1980-Present | Plot surveys | flowering and nonfloweri ng woody plants | fruiting, ripe fruit, seeds, open flowers | [ForestGEO](https://forestgeo.si.edu/) |
| --- | --- | --- | --- | --- | --- | --- | --- |
| Chinese Phenological Observation Network | China | China | 1963-2020 | Mixed | nonflowering plants | leafing, leaf color | [CPON](https://www.scidb.cn/en/detail?dataSetId=f469b05899734a89a813e45afb4a207a) |
| ClimateWatch | Australia | Australia, Vanuatu, New Zealand | 2009-Present | Crowd-sourced herbaria -- app-based | gymnosper ms, angiosper ms | Varies | [ClimateWatch](https://www.climatewatch.org.au/about) |
| e-phenology | Brazil | Brazil | 2014-2019 | Ground-based digital cameras | Varies | green-on, green-off, peak greeness | [e-phenology](https://ui.adsabs.harvard.edu/abs/2014EGUGA..1612020M/abstract) |
| PhenoCam |  | United States, Canada, Belize, Costa Rica, Panamá, Colombia, Brasil, Chile, Madagascar, DR Congo, Kenya, Europe, Israel, China, Tasmania AU, New Zealand | 2008-Present | Ground-based digital cameras | Varies | green-on, green-off, peak greenness | [PhenoCam](https://phenocam.nau.edu/webcam/) |
| Pheno-eye Japan | Japan | Japan, Russia, United States, Thailand, South Korea, France, Indonesia, United Kingdom, China, Germany, Egypt., Malaysia, Mongolia | 2003-Present | Ground-based digital cameras | Varies | green-on, green-off, peak greenness | [PEN](http://www.pheno-eye.org/) |
| Global Biodiversity Information Facility | Denmark | Global | Varies | Mixed | Varies | Varies | [GBIF -](https://www.gbif.org/dataset/search?q)  [datasets](https://www.gbif.org/dataset/search?q) |

| Global Learning and Observations to Benefit the Environment | United States | United States, Argentina, Europe, South Korea, Israel, Saudi Arabia, Japan, Thailand, Russia, South Africa, Philippines, Cameroon, Kyrgyzstan, Taiwan | 1998-Present | Community Science | deciduous trees, forb, shrubs, | Green-up, Green- down, First leaf (L, C), Full leaf (L, C), First bloom (L, C), full bloom (L, C), end of bloom (L, C), First flowering (G), general flowering (G), end of flowering (G) | [GLOBE](https://www.globe.gov/) |
| --- | --- | --- | --- | --- | --- | --- | --- |
| GLobal Observation Research Initiative in Alpine environments | Austria | Canada, United States, Greenland, Iceland, Colombia, Ecuador, Peru, Chile, Kenya, Uganda, Europe, Japan, Taiwan, Australia, New Zealand, Iran | Varies | Plot surveys | vascular plants | Varies | [GLORIA](https://gloria.ac.at/home) |
| International Phenological Gardens of Europe | Germany | Czech Republic, Estonia, Germany, Italy, Slovakia, Turkey, USA | Varies | Plot surveys | deciduous trees, forb, shrubs, | sprouting of leaves. beginning of leaf unfolding, bud burst, beginning of flowering/blossomin g, full flowering/blossom, end of flowering/blossom, first ripe fruit, fruit ripe for picking, coloring of leaves, falling of leaves | [IPG](https://ipg.ku.de/en/) |

| Indigenous weather knowledge | Australia | Australia | Varies | Traditional Ecological Knowledge | forbs, deciduous trees, graminoids  ,  deciduous shrubs, | Varies | [IWK](http://www.bom.gov.au/iwk/) |
| --- | --- | --- | --- | --- | --- | --- | --- |
| International co-operative programme on assessment and monitoring of air pollution effects on forests | NA | France, Belgium, Germany, Italy, Spain, Luxembourg, Hungary, Romania, Slovenia | 2018-2023 | Plot surveys | evergreen trees, deciduous trees | Closed buds, leaf appearance, leaf unfolding, needle appearance, needle unfolding, flowering, autumn coloring, damage | [ICP](http://icp-forests.net/) |
| International Long-term Ecological Research Network | NA | Australia, Austria, Belgium, Brazil, Bulgaria, Chile, China, Czech Republic, Finland, France, Germany, Israel, Italy, Japan, Netherlands, Poland, Portugal, Slovenia, South Africa, Spain, Sweden, Switzerland, United Kingdom, United States | Varies | Plot surveys | Varies | Varies | [ILTER](https://www.ilter.network/network/global-coverage) |
| Ireland's National Phenology Network | Ireland | Ireland | 2011-2023 | Community Science | evergreen trees, deciduous trees, deciduous shrubs | leaf unfolding, May shoot, Flowering, St. John's sprout, ripe fruits, autumn colouring, leaf fall | [IE-NPN](https://www.epa.ie/publications/research/climate-change/irelands-national-phenology-network-ie-npn.php) |
| Italian Phenology Network | Italy | Italy | 1986-2000 | Community Science | Varies | Varies | [IPHEN](https://www.researchgate.net/publication/236915807_Erratum_to_IPHEN-a_real-time_network_for_phenological_monitoring_and_modelling_in_Italy) |

| Japan Phenology Monitoring Network | Japan | Japan | 2021-2023 | Community Science | evergreen trees, deciduous trees, evergreen shrubs, deciduous shrubs, forbs, | flowering, full bloom, germination, leaf fall, blossoming, autumn coloring | [JPMON](https://adaptation-platform.nies.go.jp/ccca/monitoring/phenology/index.html) |
| --- | --- | --- | --- | --- | --- | --- | --- |
| USGS Remote Sensing Phenology | United States | United States | 2003-2023 | Satellite | Varies | green-on, green-off, peak greeness | [USGS-RSP](https://www.usgs.gov/special-topics/remote-sensing-phenology) |
| Journey North | United States | Canada, United States | 1997-2023 | Community Science | forbs, deciduous trees | first spotting, leaf out, | [Journey North](https://journeynorth.org/sightings/) |
| NatureWatch | Canada | Canada | 1995-2023 | Community Science | forbs, deciduous trees, deciduous shrubs, evergreen trees | First bloom, mid bloom, leafing out, | [PlantWatch](https://www.naturewatch.ca/plantwatch/download-data/) |
| Nature's Calendar | United Kingdom | United Kingdom | 2015-present | Community Science | grasses, trees, shrubs, forbs | Budburst, first leaf, first flower, fruit ripe, first tint, full tint, leaf fall, bare tree | [Nature's](https://naturescalendar.woodlandtrust.org.uk/) [Calendar](https://naturescalendar.woodlandtrust.org.uk/) |
| The Australasian Virtual Herbarium | Australia & New Zealand | Australasia | Varies | Herbaria | Varies | flower, leaf, buds, fruit | [AVH](https://avh-chah-org-au.wv-o-ursus-proxy02.ursus.maine.edu/about/) |

| South Africa National Biodiversity Institute | South Africa | South Africa | Varies | Herbaria, Satellite | Varies | flower, leaf, buds, fruit, green-on, green-off, peak greenness | [SANBI](https://www.sanbi.org/) |
| --- | --- | --- | --- | --- | --- | --- | --- |
